# Novel dynamic syndesmotic stabilization system improves anteroposterior and axial translation in distal tibiofibular joint

**DOI:** 10.1007/s00264-025-06706-x

**Published:** 2025-12-06

**Authors:** Firas Souleiman, Ivan Zderic, Torsten Pastor, Dominic Gehweiler, Boyko Gueorguiev, Jessica Galie, Todd Kent, Andrew Sands, John Shank, Matthew Tomlinson, Tim Schepers, Michael Swords

**Affiliations:** 1 Department of Orthopaedic, Trauma and Plastic Surgery, University Hospital Leipzig, Leipzig, Germany; 2 AO Research Institute Davos, Davos, Switzerland; 3 Department of Orthopedic and Trauma Surgery, Lucerne Cantonal Hospital, Lucerne, Switzerland; 4 Medical Faculty, University of Zurich, Zurich, Switzerland; 5 Johnson & Johnson MedTech, New Brunswick, NJ, USA; 6 Lower Manhattan Hospital, NewYork–Presbyterian Hospital, New York, USA; 7 Orthopaedic Centers of Colorado, Colorado Springs, CO, USA; 8 The Orthopaedics Clinic, Auckland, New Zealand; 9 Trauma Unit, Amsterdam UMC, Amsterdam, Netherlands; 10 Michigan Orthopedic Center, University of Michigan Health-Sparrow, Lansing, MI, USA

**Keywords:** Length-unstable syndesmotic injury, Syndesmosis, Syndesmotic pronation-eversion injury, Reconstruction, Computed tomography, Biomechanics, Screw-suture implant, Suture-button device

## Abstract

**Background:**

The quest for optimal treatment of acute distal tibiofibular syndesmotic disruptions is still in full progress. Using suture-button repair devices is one of the dynamic stabilization options, however, they may not be always appropriate for stabilization, for example in length-unstable syndesmotic injuries. The aim of this biomechanical study was to investigate whether a novel screw-suture implant addresses such issues compared to suture-button implants while preserving dynamic capabilities.

**Methods:**

Eight pairs of human cadaveric lower legs were injured by complete syndesmosis and deltoid ligaments cuts, and reconstructed using a screw-suture (FIBULINK, Group 1) or a suture-button (TightRope, Group 2) implant for syndesmotic stabilization, placed 20 mm proximal to the tibia plafond. Following, all specimens were biomechanically tested over 5000 cycles under combined 1400 N axial and ± 15° torsional loading. Anteroposterior, axial/vertical, mediolateral and torsional movements at the distal tibiofibular joint level were evaluated biomechanically via optical motion tracking.

**Results:**

Anteroposterior and axial/vertical movements were significantly smaller and maintained over the cycles in Group 1 compared with Group 2 (*p* < 0.001). No further significant differences were identified between the groups (*p* ≥ 0.318).

**Conclusion:**

Although both implant systems demonstrate ability for stabilization of unstable syndesmotic injuries, the screw-suture reconstruction provides better anteroposterior and axial/vertical stability of the distal tibiofibular joint, and maintains it over time under dynamic loading in a cadaveric study design. Therefore, it could be considered as a valid option for treatment of syndesmotic disruptions with length-unstable fibula.

**Level of evidence/ study design:**

Level V, Controlled Laboratory Study.

## Introduction

The incidence of syndesmosis injuries in ankle sprains has been reported between 20 and 40%, increasingly arising in high-impact sports or in combination with an ankle fracture [1–3]. In case of an unstable syndesmotic injury, anatomic reduction and stabilization is recommended to gain better clinical results and prevent instability, pain, and secondary degenerative changes [4–7]. The principle in operative treatment of ankle fractures with associated syndesmotic injuries is to restore fibula length, rotation, and then correct the anatomic alignment in the fibular notch [8]. This reduction is maintained by the syndesmotic fixation [9]. However, there is an ongoing debate regarding the preferred stabilization system and its superiority [4, 10–15]. Three-dimensional (3D) studies on the distal tibiofibular joint demonstrated that the relative movement of the fibula around the tibia depends on the foot position [16]. A recent study reported very good results for both static-screw and dynamic suture-button systems with significantly faster return to sport activities for the latter [4]. In addition, lower rates of malreduction were reported for suture-button devices. It has been reported that, due to the dynamic stabilization, “the flexible nature of fixation” has the ability to compensate for intraoperative malreduction [17, 18]. 

However, some disadvantages of the suture-button device have been reported along with its advantages. Medial soft-tissue disruptions with pain, breakage or retracting/loosening of the button to the medullary space of the tibia were reported as limitations [13]. As concluded by Riedel and colleagues, when treating a length-unstable fibula fracture (e.g. Weber-C or Maisonneuve), dynamic stabilization can result in accidental malreduction with fibular shortening due to the unstable proximal fracture situation [8]. The suture-button device is described as being unable to hold the syndesmosis joint stable. One cause is that the drill used to insert the suture-button device is determined by the button and not by the suture. The drill used to pass the suture-button is 3.7 mm, whilst the eight strands of suture are significantly thinner, allowing for motion in the drilled tunnel. As a result, the tensioned suture has movement in the drill holes, which is undesirable in case of length-unstable fractures. In order to improve stability in length-unstable injury patterns, a combination of dynamic suture-button devices and static screws is recommended for stabilization at least six weeks after operation [8]. 

**Fig. 1:**
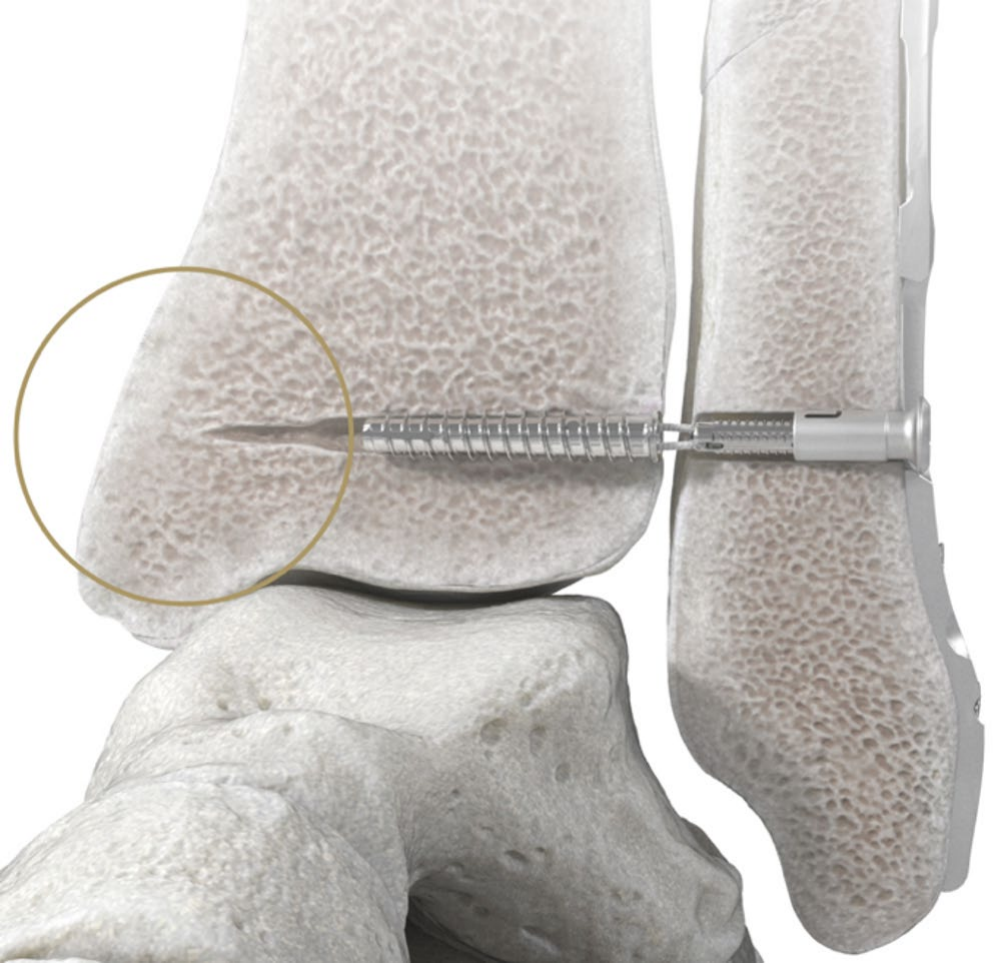
Visualization of the novel syndesmotic screw-suture repair system FIBULINK ^TM^ Syndesmosis Repair System (Johnson & Johnson MedTech, Zuchwil, Switzerland). No medial soft tissue disruption occurs during implantation due to the intramedullary tibial screw fixation.

To minimize the disadvantages of the suture-button system mentioned above, a new type of screw-suture implant (Fig. 1) was developed that combines the advantages of the bony anchorage of a screw with the dynamic properties of a suture. For surgical implantation, a single lateral approach is required since the implant is fixed intramedullary.

A torque limiter has been built into the tensioning knob to prevent applying too much tension to the system. So far, this novel implant has never been investigated biomechanically in human cadaveric studies against other established dynamic implants.

Therefore, the aim of this study was to investigate biomechanically the competence of the novel screw-suture device in comparison to the current standard suture-button implant in constraining syndesmosis motion and fibula length in a human cadaveric model.

## Materials and methods

### Specimens & preparation

Sixteen paired fresh-frozen (-20 °C) human cadaveric lower legs from eight male and two female donors aged 70.1 ± 9.3 (mean ± standard deviation, SD) years (range 54–82 years) with no visible preexisting pathology, trauma or surgery were used for this controlled laboratory study (Level V). All donors gave their informed consent inherent within the donation of the anatomical gift statement during their lifetime. The specimens were thawed 24 h before preparation and testing, and embedded at the level of the proximal tibia in polymethylmethacrylate (PMMA; SCS-Beracryl, Suter-Kunststoffe AG, Fraubrunnen, Switzerland) with intact ankle ligaments and interosseous membrane. During embedding, care was taken to exclude the proximal tibiofibular joint so that the fibula could move in all directions.

### Injury model

To destabilize the distal tibiofibular joint, the syndesmotic ligament complex (anterior inferior tibiofibular ligament [AITFL], ligamentum interosseum [IOL], posterior inferior tibiofibular ligament [PITFL]) and the distal 10 cm of the interosseous membrane were cut [19]. To achieve this, skin incisions were made on the lateral malleolus without damaging the remaining soft tissues. To visualize the standardized instability of the distal tibiofibular joint, a spreader was used to diverge the tibia and fibula at least 1 cm from each other. This ligament injury model is equivalent to Grade 3 of the West Point Ankle Grading System and corresponds to pronation-eversion injuries type III and IV as well as supination-eversion injuries type IV according to Lauge-Hansen [20, 21]. In combination with the manner of embedding—avoiding additional restrictions of the proximal tibiofibular joint movements—the injury model was designed to simulate a length-unstable fibula situation with injury of the syndesmotic complex without adding fibula fractures to avoid additional bias. Following the Lauge-Hansen classification of high fibula/Maisonneuve fractures, as well as new studies describing a high co-incidence of severe syndesmotic injuries with deltoid ligament lesions of up to 81%, the complete deltoid ligament complex with its superficial and deep layers was cut [21, 22]. 

### Surgical stabilization

For anatomic stabilization of the distal tibiofibular joint, the lower legs of each donor were split into two groups with equal distribution of left and right anatomical sites each. The specimens in Group 1 were stabilized with the novel screw-suture implant (FIBULINK ^TM^ Syndesmosis Repair System, Johnson & Johnson MedTech, Zuchwil, Switzerland), whereas the surgical stabilization in Group 2 was achieved using a conventional suture-button implant (Syndesmosis TightRope^®^ XP Implant System, Arthrex, Naples, FL, USA). To ensure an anatomical reduction, Kirschner (K-) wire holes were pre-drilled in the intact state. These holes were used to ensure anatomical reduction for subsequent implant positioning. Both systems were implanted singularly 20 mm proximal to the tibial plafond, without applying any further stabilization procedures and following the manufacturers’ guidelines [23, 24]. 

### Biomechanical testing

**Fig. 2:**
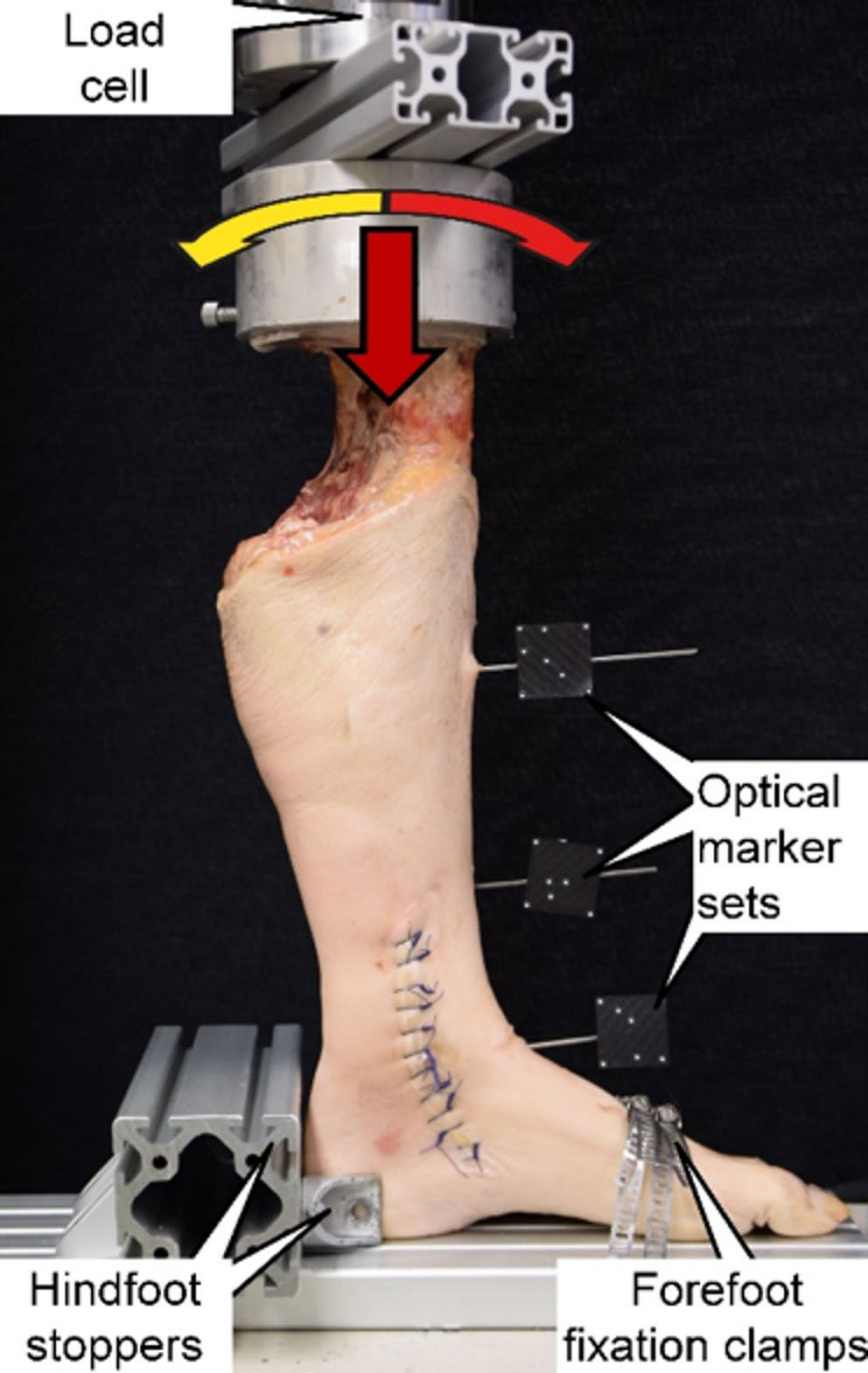
Setup with a specimen mounted for biomechanical testing. Vertical arrow denotes axial loading direction. Curved arrows denote torsional loading direction

Non-destructive dynamic biomechanical testing was performed on a servo-hydraulic bi-axial material testing machine (MiniBionix 848.2, MTS Systems Corp., Eden Prairie, MN, USA) equipped with a 5 kN/100 Nm load cell (HBM, Darmstadt, Germany). The legs were mounted and tested in vertical position with the foot sole being supported by the machine base in neutral position, simulating weight-bearing in mid-stance position. For this purpose, the proximal embedding was fixed to the machine transducer via a custom holder. Whereas the forefoot was fixed with metal clamps to the machine base, the hindfoot was restricted in sideways and backward movements by means of stoppers positioned dorsally (Fig. 2).

This setup allowed tight anchoring of the foot without restriction the movements in the ankle joint. The fibula was not fixed proximally in PMMA to avoid additional restrictions of the proximal tibiofibular joint movements.

The test protocol comprised complex axial and torsional cyclic sinusoidal loading at 0.5 Hz over 5000 test cycles. Whereas the torsional loading was applied between 15° internal and 15° external rotation in angle control, axial compression along the machine axis was applied within the range 50 N valley load and 1400 N peak load. Thereby, peak internal/external rotation loading was synchronized with peak/valley axial loading, respectively. Whereas the magnitude of the valley load represented the muscular tension of the lower leg muscles, the peak load simulated two-fold body weight. To analyze the exact relative movements of the bones at the level of the implant, monitoring was performed via motion tracking using a stereographic optical camera system (Aramis SRX, GOM GmbH, Braunschweig, Germany) operating at 12-megapixel resolution and 0.004 mm maximum acceptance error. For this purpose, 2 marker sets were attached to the fibula and tibia using K-wires and a further virtual marker was registered at the level of the implant. All movements were tracked in six degrees of freedom.

### Data acquisition and analysis

The motion tracking data derived during biomechanical testing were processed to analyze the magnitude of the displacement between the tibia and fibula at the implant level in anteroposterior (AP), axial (vertical/superior-inferior) and mediolateral (ML) direction, along with the total displacement and rotation between them at the beginning of the cyclic test (cycle 0) and then after 1000, 2000, 3000, 4000 and 5000 cycles. For this purpose, an anatomical cartesian coordinate system was generated by proper alignment of the marker sets.

Statistical analysis was performed with SPSS software package (V.26, IBM SPSS, NY, USA). Normal distribution of the data was screened and confirmed with Shapiro-Wilk tests. The motion tracking outcome measures were pooled among the investigated time points for each parameter of interest and group separately. Based on this, differences between the groups were assessed with Wilcoxon-Signed Rank test for each parameter separately. Level of significance was set to 0.05 for all statistical tests.

## Results

**Fig. 3:**
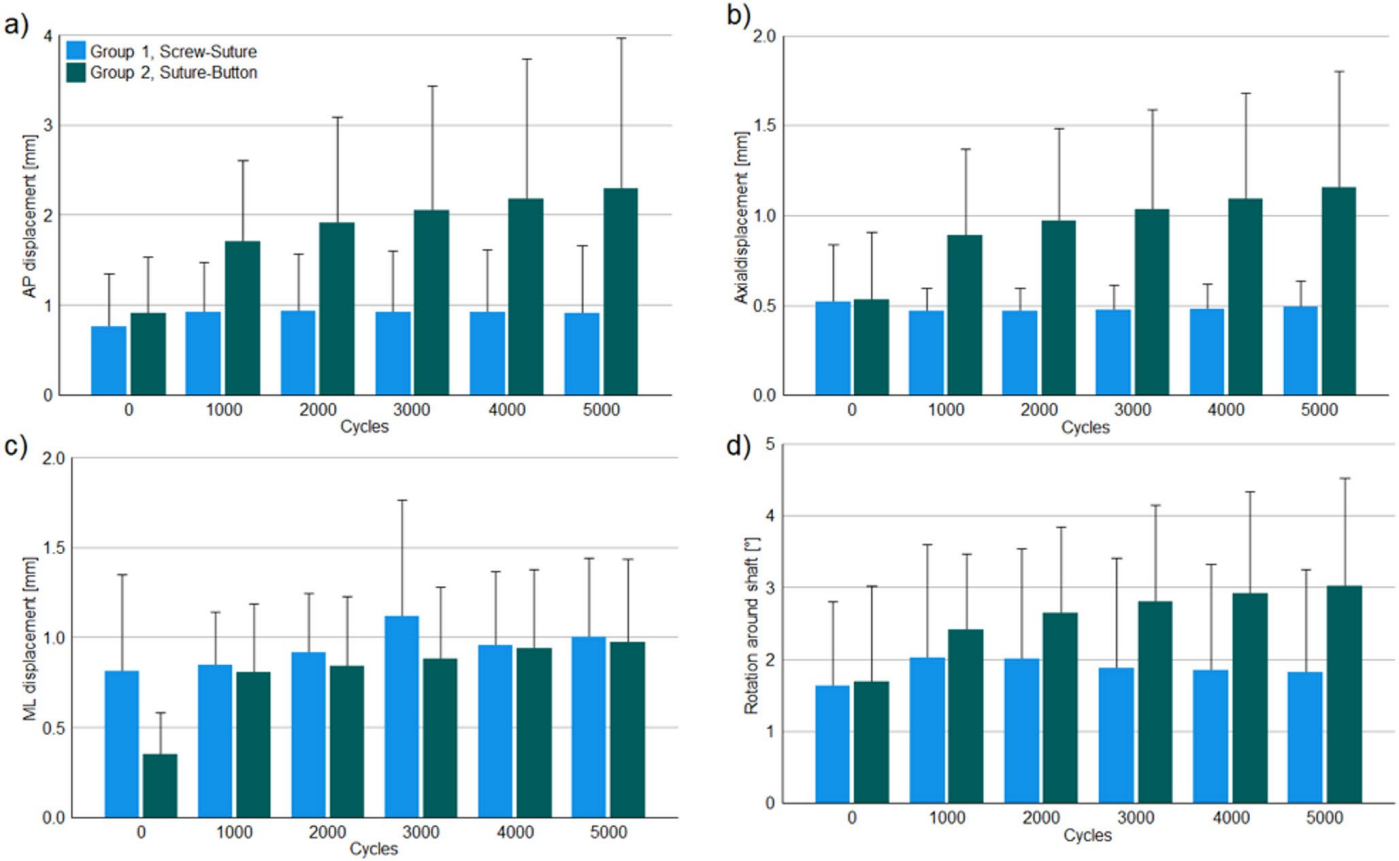
Outcome measures during biomechanical testing of the screw-suture (FIBULINK TM Syndesmosis Repair System, Johnson & Johnson MedTech, Zuchwil, Switzerland; Group 1) and suture-button (Syndesmosis TightRope XP Implant System, Arthrex, Naples, FL, USA; Group 2) implants: anteroposterior (AP) (a), axial (b) and mediolateral (ML) (c) displacements, and rotation (d) between the tibia and fibula presented in terms of mean value and standard deviation.

Explorative data of the outcome measures under peak loading conditions during biomechanical testing are summarized in Table 1 and presented in Fig. 3.

Total, AP and axial/vertical displacements under peak loading were associated with significantly smaller values in Group 1 versus Group 2 (*p* ≤ 0.027).

The screw-suture implant demonstrated minimal to no accidental AP and axial displacements over the course of 5000 cycles, while the suture-button implant showed an unintended increase of the AP and axial displacements from 0.902 mm to 2.289 mm and from 0.533 mm to 1.156 mm on average over 5000 cycles, respectively.

No significant differences were identified between the groups for the ML displacement and rotation, *p* ≥ 0.318.

**Table 1:**
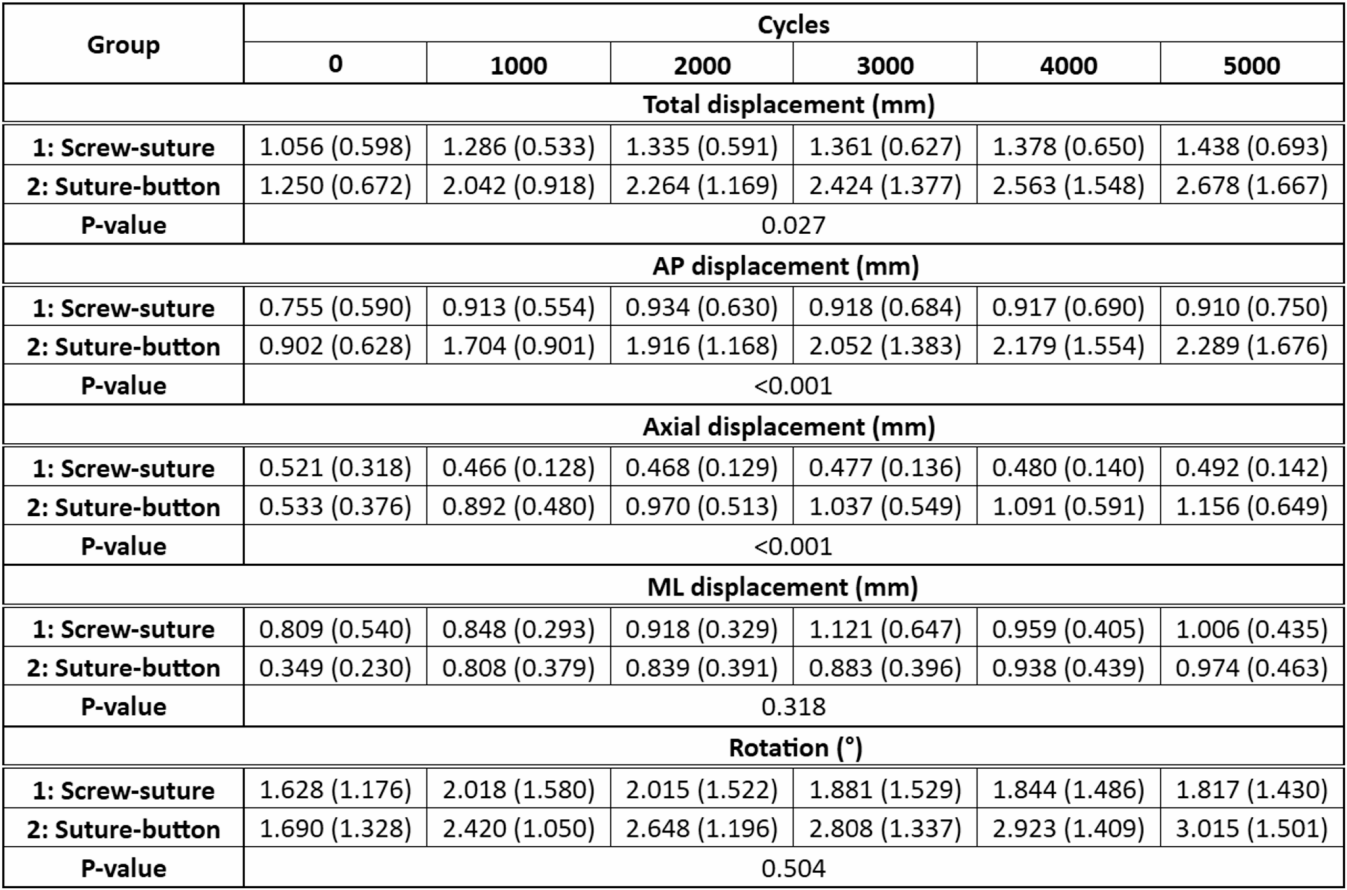
Outcome measures during biomechanical testing of the screw-suture (FIBULINK TM Syndesmosis Repair System, Johnson & Johnson MedTech, Zuchwil, Switzerland; Group 1) and suture-button (Syndesmosis TightRope XP Implant System, Arthrex, Naples, FL, USA; Group 2) implants: total, anteroposterior (AP), axial and mediolateral (ML) displacements, and rotation between the tibia and fibula presented in terms of mean value and standard deviation together with the p-values from the statistical comparison between the groups.

## Discussion

The aim of this study was to investigate biomechanically the competence of the novel screw-suture device in comparison to the current standard suture-button implant in constraining syndesmotic motion and fibula movement in an injured human cadaveric model. The main findings are that the novel screw-suture implant provided significantly more axial/vertical (average: 0.4 mm to 0.5 mm) and sagittal (average: 0.8 mm to 1.0 mm) stability and maintains it over time under dynamic loading, compared to the established suture-button system while preserving dynamic capabilities. One possible explanation could be that the screw-suture implant combines both positive effects of better screw anchoring in bone without movement of the suture in the bone canal, while providing the dynamic character due to the mobile suture. In contrast to the suture-button system where the buttons are anchored extramedullary, the screws in the screw-suture device are anchored intraosseous in both the tibia and the fibula (tibia screw and fibula link). This reduces the working length of the suture bridge in the screw-suture implant (connection between the screws and the suture) and improves the stability compared to the suture-button system. Over the cycles, the suture-button construct has slightly yielded and cut into the tibial and fibular bone proximal/distal as well as anterior/posterior in the drill tunnel (like ‘wipers’). This resulted in poorer axial and anteroposterior stability of the suture-button implant over the course of cyclic loading, resulting in a subsequent loss of the initially implanted joint position, which does not correspond to the desired dynamic character of the implant.

This appears to be the biomechanical reason for the loss of reposition reported by Riedel and colleagues in single implantation of a suture-button implant in the case series of length-unstable fibula fractures [8]. As a recommended solution, they describe how the suture-button system is combined with a screw to achieve a stable distal tibiofibular joint until the syndesmotic ligaments have healed [8]. Later removal of the screw requires additional surgery [25]. Based on the biomechanical findings of the current study, the novel hybrid screw-suture implant could possibly provide sufficient stability as a single implant to stabilize the syndesmosis in case of length- unstable fibula fractures. As known from other studies, misalignment of the distal tibiofibular joint leads to increased tibiotalar pressure with increased risk of osteoarthritis and pain [26–28]. 

The injury model in the present work was adapted to existing test setups to achieve most physiological test conditions of a severe syndesmotic injury with a length-unstable fibula. As the deltoid ligament also contributes to syndesmotic stability acting as a restraint against lateral shift of the talus, it was also cut in the injury model [29, 30]. Furthermore, there is a high co-incidence of a severe syndesmotic injury with an additional deltoid ligament lesion observed in up to 81% of the cases [22]. Both the superficial and deep layers were cut in order to standardize the injury to the deltoid ligament. For biomechanical testing, other studies performed torsional loading in external and internal rotation of the ankle under torque control of 7.5 Nm [31, 32]. Since in preliminary pilot tests the observed magnitude of rotation performed under torque control was dependent on the cadaveric anatomy of the different specimens, the torsional loading in the current study was applied between 15° internal and 15° external rotation in angle control. The cutting of the AITFL, PITFL, IOL and interosseous membrane was performed as in other existing biomechanical studies to injure all parts of the syndesmotic complex [33–35]. 

This study is the first to biomechanically investigate the novel dynamic screw-suture implant in a human cadaveric model comparing it with an established suture-button implant in the same test setup and showing that the screw-suture implant could be of great benefit for treatment of length-instable fibula fractures without the need of using an additional screw to maintain reduction—as in some cases with suture-button implants—and without the necessity to remove this screw afterwards.

Knowing that the distal tibiofibular joint position is a complex dynamic system, the strength of the present study is the combination of a dynamic non-destructive biomechanical test setup coupled with motion tracking to identify different implant characteristics.

In general, the standard suture-button implant cannot be controlled as precisely in terms of over-tensioning as the new screw-suture implant handled via the screw-thread control.

The present study has some limitations inherent to all human cadaveric biomechanical studies (no implementation of active muscle tension, static-joints conditions, simulation of a gait cycle under assumptions). Moreover, with regard to the motion tracking analysis, no baseline comparison between intact and injured condition of the specimens was performed. This was not done because the authors considered that the rather aggressive biomechanical test protocol would have caused damage to the lower extremities during the baseline measurements before starting the controlled ligament injuries and surgical stabilization. Further, a limited number of specimens was considered and the study was performed on lower extremities from advanced-age donors. It can be assumed that in the clinical practice the bone quality in affected young patients with distal tibiofibular ligament lesions would be better compared to the one of the used cadaveric specimens. Therefore, expected tension loosening/wiper effect of the suture-button implant could be lower in better bone quality. On the other hand, the novel screw-suture implant would possibly provide less displacement in poorer bone quality, thus being advantageous for applications in older patients.

A major advantage of this study is the precise measurement of movements at the implant level via motion tracking and its coupling with the number of corresponding loading cycles, along with the implementation of a complex test setup. Overall, it can be summarized that the chosen study design had sufficient power to crystalize differences between the two dynamic implants. The novel screw-suture implant is another dynamic tool that appears more stable in axial/vertical direction and could therefore be used for treatment of length-unstable syndesmotic injuries. For a further confirmation the study should be repeated by using a Maisonneuve fracture model. The clinical application of the novel screw-suture implant should be investigated in future studies to answer the crucial question of how much stability is required for ideal syndesmotic ligament healing, because this still remains unclear. Solving this issue, as well as the clinical application of the novel screw-suture implant in length-unstable fibula fractures, seems exciting based on the current findings.

## Conclusion

Although both implant systems demonstrate ability for stabilization of unstable syndesmotic injuries, the screw-suture reconstruction provides better anteroposterior and axial/vertical stability of the distal tibiofibular joint, and maintains it over time under dynamic loading in a cadaveric study design. Therefore, it could be considered as a valid option for treatment of syndesmotic disruptions with lengthunstable fibula.

## Data Availability

No datasets were generated or analysed during the current study.
